# Endothelial Activation in *Orientia tsutsugamushi* Infection Is Mediated by Cytokine Secretion From Infected Monocytes

**DOI:** 10.3389/fcimb.2021.683017

**Published:** 2021-07-22

**Authors:** Wiwit Tantibhedhyangkul, Sutthicha Matamnan, Asma Longkunan, Chawikan Boonwong, Ladawan Khowawisetsut

**Affiliations:** ^1^ Department of Immunology, Faculty of Medicine Siriraj Hospital, Mahidol University, Bangkok, Thailand; ^2^ Graduate Program in Immunology, Faculty of Medicine Siriraj Hospital, Mahidol University, Bangkok, Thailand; ^3^ Research Division, Faculty of Medicine Siriraj Hospital, Mahidol University, Bangkok, Thailand; ^4^ Department of Parasitology, Faculty of Medicine Siriraj Hospital, Mahidol University, Bangkok, Thailand

**Keywords:** *Orientia tsutsugamushi*, scrub typhus, endothelial cell, chemokine, tumor necrosis factor (TNF)

## Abstract

Scrub typhus, caused by *Orientia tsutsugamushi*, is a common systemic infection in Asia. Delay in diagnosis and treatment can lead to vasculitis in the visceral organs and other complications. The mechanisms that drive endothelial activation and the inflammatory response in *O. tsutsugamushi* infection remain unknown. In addition, the interaction between monocytes and endothelial cells is still unclear. Here we demonstrate that *O. tsutsugamushi*-infected human dermal microvascular endothelial cells produced moderate levels of chemokines and low levels of IL-6 and IFN-β, but not TNF or IL-1β. Recombinant TNF and cytokine-rich supernatants from infected monocytes markedly enhanced chemokine production in infected endothelial cells. We also show that TNF and monocyte supernatants, but not *O. tsutsugamushi* infection of endothelial cells *per se*, upregulated the endothelial cell surface expression of ICAM-1, E-selectin, and tissue factor. This finding was consistent with the inability of *O. tsutsugamushi* to induce cytokine secretion from endothelial cells. The upregulation of surface molecules after stimulation with monocyte supernatants was significantly reduced by neutralizing anti-TNF antibodies. These results suggest that endothelial cell activation and response are mainly mediated by inflammatory cytokines secreted from monocytes.

## Introduction

Scrub typhus, caused by *Orientia tsutsugamushi* (OT), is a common cause of acute undifferentiated febrile illness in endemic areas including Asia and Northern Australia (Suttinont et al., 2006; Tantibhedhyangkul et al., 2017b). Since new cases of scrub typhus have been reported in South America and Africa, the disease has been recognized as an emerging infection (Thiga et al., 2015; Weitzel et al., 2016; Weitzel et al., 2019). The symptoms of scrub typhus are non-specific and cannot be distinguished from other systemic infections. When treatment is delayed, patients may develop interstitial pneumonitis, meningoencephalitis, disseminated intravascular coagulation, and death (Seong et al., 2001). Moreover, various complications in different organ systems resulting from vasculitis have been reported in patients (Jeong et al., 2007; Lee et al., 2016).

Both *Rickettsia* and *Orientia* spp. have tropism for endothelial cells (ECs) (Valbuena and Walker, 2009), whereas OT also invades monocytes, dendritic cells and tissue macrophages (Moron et al., 2001; Tantibhedhyangkul et al., 2011; Paris et al., 2012b). The invasion of these rickettsial organisms into ECs results in vascular injury accompanied by perivascular mononuclear infiltration in visceral organs (Moron et al., 2001). The vascular damage and inflammation can cause complications. Previous studies have demonstrated that OT induces cytokine and chemokine transcript expression in ECs such as human dermal microvascular endothelial cells (HMECs) and human umbilical vein endothelial cells (HUVECs) (Cho et al., 2001; Ge et al., 2019; Mika-Gospodorz et al., 2020), but the protein expression of these mediators has not been studied. EC activation markers are also increased in sera of patients with scrub typhus (Paris et al., 2012a). Animal model studies have demonstrated the modulation of angiopoietin (Ang1 and Ang2) by OT infection and suggested that EC activation and dysfunction in visceral organs underlie the pathology of scrub typhus (Soong et al., 2014; Shelite et al., 2016; Soong et al., 2017; Trent et al., 2020). Moreover, OT infection of monocytes results in high levels of cytokine expression and secretion (Tantibhedhyangkul et al., 2011), which may lead to systemic inflammation. However, the effects of cytokines secreted from monocytes on ECs during OT infection have not been studied. Indeed, inflammatory cytokines, particularly TNF and IL-1, are well known to promote EC activation (Grignani and Maiolo, 2000). Therefore, we questioned whether EC activation in OT infection is mediated by infected ECs *per se* or by the cytokines secreted from infected monocytes.

This study of human dermal microvascular endothelial cells (HMECs) demonstrates that OT infection prompts expression and secretion of chemokines, but not cytokines TNF and IL-1β. Endothelial cell activation (ICAM-1 and E-selectin upregulation) is mediated by TNF and supernatants from infected monocytes, but not by direct OT infection of HMECs. Cytokines secreted from monocytes also induce endothelial expression of tissue factor, an essential initiator of the extrinsic coagulation pathway (Grover and Mackman, 2018). The poor induction of endothelial cell activation and cytokine secretion by OT infection *per se* may represent the organism’s host evasion strategy.

## Materials and Methods

### Cultivation of Orientia tsutsugamushi

OT strain Karp was propagated in L929 mouse fibroblast cultures with RPMI 1640 and 5% fetal bovine serum (Gibco Thermo Fisher Scientific, Waltham, MA). When the heavily infected L929 cells showed a cytopathic effect, they were disrupted by repeated passage through a 25-gauge needle and syringe. The cell suspension was centrifuged at 400 × *g* for 5 min to remove the cell pellet. Supernatants containing extracellular OT were resuspended in RPMI 1640 with 7% DMSO and stored at –80°C. Infectivity was measured as infected cell count units (ICU), as described previously (Tantibhedhyangkul et al., 2011). The ratio of ICU to cells is comparable to the multiplicity of infection (MOI).

### Cultivation of Primary Human Dermal Microvascular Endothelial Cells (HMECs) and Human Monocytes

Commercially available HMECs (CSC 2M1; Cell Systems, Kirkland, WA) were grown in 0.5% gelatin-coated T75 in a humidified 5% CO_2_ incubator at 37°C. Culture medium was MCDB131 medium (Sigma-Aldrich, St. Louis, MO) supplemented with 10% fetal bovine serum, heparin 0.75 U/ml, hydrocortisone 0.5 µg/ml, epidermal growth factor (EGF) 10 ng/ml, insulin-like growth factor (IGF-1) 15 ng/ml, fibroblast growth factor (FGF-basic) 5 ng/ml, and vascular endothelial growth factor (VEGF165) 2.5 ng/ml (ImmunoTools, Friesoythe, Germany). The medium was changed every other day. When HMEC layers were nearly confluent, cells were subcultured using 0.05% Trypsin/EDTA. Some detached cells were subjected to experiments performed on the following day.

Peripheral blood mononuclear cells (PBMCs) were isolated from buffy coats of healthy blood donors (provided by blood bank) using lymphocyte separation medium (Biowest, Nuaille, France). The protocol was approved as a “Research with Exemption” category by Siriraj Institutional Review Board. To obtain adherent monocytes, PBMCs were left to adhere to 24-well plates for 90 minutes, and non-adherent lymphocytes were removed by washing with PBS. The purity of adherent monocytes was higher than 90%, as determined by CD14 expression using flow cytometry.

### Cell Stimulation Experiments

HMECs were infected with OT at an ICU-to-cell ratio of 20:1 for 1 hour, as described previously (Ge et al., 2019). The infected cells were washed with PBS and maintained for 4 or 8 h (mRNA expression) or 18 h (cytokine secretion), as indicated. For other studies, HMECs were stimulated with recombinant TNF 10 ng/ml (ImmunoTools) or supernatants from infected monocytes (diluted 1:15 with MCDB131) for 4 h (mRNA expression) or 18 h (cytokine secretion).

Monocytes were infected with OT at an ICU-to-cell ratio of 20:1 for 1 hour. We showed that an incubation time of 1 hour was sufficient for host cells (both HMECs and monocytes) to internalize OT organisms **(** **Supplementary Figure 1** **)**. Supernatants were collected at 18 h, centrifuged at 10,000 × *g* for 10 min, and filtered through 0.2 µm syringe filter, then stored at –80°C. The absence of infectivity was confirmed by indirect immunofluorescence staining, as described previously (Tantibhedhyangkul et al., 2011).

### Cytokine/Chemokine Expression and Secretion by qRT-PCR and ELISA

Total RNA was extracted from HMEC cell lysates using the GenUP™ total RNA kit (Biotechrabbit, Hennigsdorf, Germany). RNA was converted to cDNA using SuperScript III reverse transcriptase (Invitrogen Thermo Fisher Scientific) and subjected to qRT-PCR using SYBR Green master mix and a CFX96™ real-time PCR detection system (Bio-rad, Hercules, CA). The fold change of the target genes relative to *GAPDH* was calculated using the 2^-ΔΔCq^ method. ELISA kits were used to measure supernatant cytokine levels of TNF, IL-6, CCL2 (MCP-1), CXCL10 (IP-10) (KOMA biotech, Seoul, Korea), and IFN-β (MyBioSource, San Diego, CA). IL-1β was determined using either the standard IL-1β ELISA kit (KOMA biotech**)** or the IL-1β ELISA high sensitivity kit (eBioscience Thermo Fisher Scientific).

### Immunoblotting of Proteins Related to Inflammasome Complex

To study protein expression of components associated with inflammasome complex, HMECs were primed or not primed with *E. coli* LPS 1 µg/ml (Sigma-Aldrich) for 6 h before infection with OT, because LPS is known to upregulate NLRP3 and pro-IL-1β protein expression (Guo et al., 2015; He et al., 2016). Cell lysates and supernatants were collected at 6–48 h post infection. LPS-primed HMECs were also stimulated with 5 mM ATP (Abcam) for 45 min and included as a control because ATP is a known inflammasome activator in macrophages (He et al., 2016). LPS-primed and OT-infected monocyte-derived macrophages (MDM) were used as a positive control for cell lysates, whereas *Salmonella*-infected MDM were used as a control for cleaved caspase-1 in supernatants because our preliminary data found that *Salmonella* triggered a higher level of cleaved caspase-1 release than OT (**Supplementary Figure 2**). To prepare cell lysates and supernatants of *Salmonella*-infected MDMs, MDMs were infected with *Salmonella* Typhimurium for one hour at an MOI of 100:1 and washed with media. Extracellular bacteria were killed with gentamicin (50 µg/ml) for 30 min. Cell lysates and supernatants were collected at 10 hours post-infection.

Cell lysates were prepared using Cell Lysis Buffer provided in Caspase-1 Assay Kit (Abcam, Cambridge, UK). Serum-free supernatants were concentrated using Amicon Ultra-0.5 ml Centrifugal Filter Unit 10kDa (Merck Millipore). Proteins were quantified by Bradford assay (Bio-Rad, Hercules, CA). Samples were mixed with 5× Laemmli buffer, heated at 95°C for 5 min, separated in 12% SDS-PAGE gels, transferred onto PVDF membranes, and blocked with 5% non-fat dry milk in PBST. Proteins were stained with rabbit anti-pro-caspase-1, anti-pro-caspase-4, anti-pro-IL-1β, anti-NLRP3, and anti-β-actin (Cell Signaling, Danvers, MA), whereas proteins in supernatants were stained with rabbit-cleaved-caspase-1 p20 (Cell Signaling) and mouse anti-IFN-β (BioLegend, San Diego, CA). Membranes were incubated with HRP-labelled secondary antibodies, reacted with Luminata™ Forte Western Chemiluminescent HRP Substrates (Merck Millipore), and visualized using ImageQuant™ LAS 4000 system (GE Healthcare, Chicago, IL).

### Surface Expression of Adhesion Molecules and Tissue Factor by Flow Cytometry

HMECs were stimulated by OT infection, TNF, or cytokines from supernatants for 24 h. The stimulated and control cells were detached with 0.5 mM EDTA in PBS with 0.5% human serum albumin (Sporn et al., 1993; Dignat-George et al., 1997), then stained with FITC anti-CD54 (ICAM-1; clone 15.2; Bio-Gems, Westlake Village, CA), PE anti-CD142 (tissue factor, clone NY2), and APC anti-CD62E (E-selectin; clone HAE-1f; Biolegend) at 4°C for 30 min. After staining, cells were washed three times, fixed in 2% paraformaldehyde for 30 min, and resuspended in PBS for flow cytometry analysis. Unstained and single-stained cells were included as controls. Surface expression was analyzed by BD FACS Calibur (BD Biosciences) and FlowJo software. We determined whether monocyte-secreted TNF mediates EC activation by pretreating HMECs with either 5 µg/ml neutralizing anti-TNF (clone # 1825; R&D systems, Minneapolis, MN) or isotype control (BioLegend) for 30 min before supernatants from OT-infected monocytes were added (dilution 1:15) for 24 h. Surface expression of ICAM-1, tissue factor, and E-selectin was analyzed by flow cytometry.

To confirm the results of surface molecule expression on detached endothelial cells, we also performed direct immunofluorescence staining of adherent HMECs in 12-well-plates using these three antibodies. After 30 min, stained cells were washed and detached using PBS/EDTA with 0.5% human serum albumin. After cell dissociation, new medium was added to stop the cell detachment. Detached cells were pelleted, washed with PBS, fixed in 2% paraformaldehyde and analyzed by flow cytometry.

### Statistical Analyses

Statistical analyses were performed using GraphPad Prism Sofware v. 5.01. The results are expressed as the mean ± SEM of three independent experiments. Statistical significance was calculated by IBM SPSS statistics v24.0 using unpaired Student’s *t*-test or paired *t*-test. *P* values less than 0.05 were considered significant.

## Results

### Chemokines and IL-6, but Not TNF or IL-1β, Are Weakly Induced by OT Infection in HMECs, and the Response Is Enhanced by Infected Monocyte Supernatants

**Figure 1:**
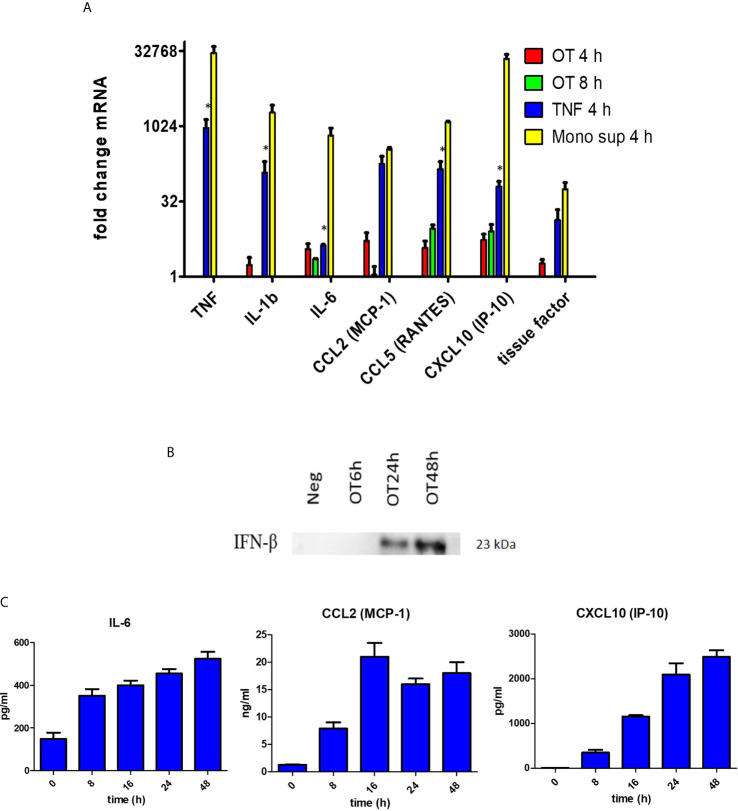
Cytokine and chemokine production by HMECs. **(A)** qRT-PCR result of *TNF, IL1b, IL6, CCL2, CCL5, CXCL10* and tissue factor mRNA expression in HMECs stimulated with different stimuli. Fold changes in gene expression levels relative to unstimulated cells were expressed as mean ± SEM of three independent experiments. Statistical significance was compared between TNF and monocyte supernatant-stimulated HMECs. *P value < 0.05. **(B)** Immunoblot of IFN-β in concentrated supernatants of OT-infected HMECs. **(C)** Cytokine and chemokine secretion from OT-infected HMECs. HMECs were infected with OT for 8 h to 48 h. IL-6 and chemokines in supernatants were analyzed by ELISA. Data are expressed as as mean ± SEM of three independent experiments.

Since endothelial cells are non-immune cells, we questioned whether the cytokine/chemokine response of HMECs is similar to or different from monocytes. We showed that OT can replicate in HMECs with a doubling time of approximately 14 hours (**Supplementary Figure 3**). Quantitative RT-PCR showed that OT-infected HMECs expressed low levels of *IL1B* and *IL6*, and *TNF* was undetectable; however, chemokines (*CCL2*, *CCL5*, and *CXCL10*) that mediate mononuclear cell migration were moderately expressed. When HMECs were stimulated with recombinant TNF (10 ng/ml) or supernatants from OT-infected monocytes (dilution of 1:15, containing approximately TNF 10 ng/ml), both cytokine and chemokine transcripts were markedly upregulated (**Figure 1A**). We also analyzed tissue factor expression, which is involved in the extrinsic coagulation pathway and is induced by inflammation in endothelial cells (Grover and Mackman, 2018). We showed that tissue factor was highly upregulated in HMECs after TNF or monocyte supernatants stimulation (**Figure 1A**).

ELISA results confirmed that HMECs secreted neither TNF nor IL-1β. Undetectable levels of TNF (<7.8 pg/ml) and IL-1β (<0.1 pg/ml) from infected HMECs were confirmed by standard and highly sensitive ELISA kits, respectively. However, unstimulated HMECs secreted IL-6, and the levels were increased by 1.5 fold after OT infection (**Table 1**). We also tried to detect IFN-β in the supernatant because previous studies have shown that it signifies infection by cytosolic pathogens, including OT (Tantibhedhyangkul et al., 2011). Although the IFN-β level (<7.8 pg/ml) was undetectable by ELISA, it was detected by immunoblot of concentrated supernatants. The IFN-β level was detected at 24 h and slightly increased at 48 h (**Figure 1B**). As expected, OT-infected monocytes secreted high levels of these cytokines, TNF being the highest.

**Table 1:** Cytokine secretion from HMECs and monocytes.

Cytokines	Levels in supernatants, mean (SEM)			
	HMECs		Monocytes	
	Neg.	Infected	Neg.	Infected
TNF (ng/ml)	ND	ND	1.45 (0.02)	161.48 (16.98)
IL-1β (ng/ml)	ND	ND	ND	11.97 (1.29)
IL-6 (ng/ml)	0.23 (0.02)	0.33 (0.01)	13.23 (1.24)	108.75 (17.02)
IFN-β (pg/ml)	ND	ND#	ND	66.5 (17.32)

ND, not detected.

#IFN-β was undetectable by ELISA but could be detected in concentrated supernatants by immunoblotting.

In contrast to cytokine secretion, HMECs secreted both CCL2 and CXCL10 at levels markedly increased by OT infection and TNF treatment. The levels of chemokine secretion from TNF-treated HMECs were significantly higher (by 7–10 fold) than OT-infected HMECs. The ability of HMECs to secrete CXCL10 after OT infection was just slightly lower than that of monocytes (**Table 2**). These findings suggest that monocytes are the primary cytokine producer, whereas both endothelial cells and monocytes can secrete high levels of chemokines to attract mononuclear leukocytes to infected sites.

**Table 2:** Chemokine secretion from HMECs and monocytes that are unstimulated, infected by OT or stimulated by TNF (10 ng/ml).

Chemokines	Levels in supernatants, mean (SEM)				
	HMECs			Monocytes	
	Neg.	OT	TNF	Neg.	OT
CCL2 (MCP-1)	6.05	16.73^*^	116.95^*^	35.25	109.33
(ng/ml)	(1.42)	(2.67)	(17.42)	(2.16)	(24.77)
CXCL10 (IP-10)	8.51	508.67^*^	4,749.57^*^	345.67	1,263.87
(pg/ml)	(0.52)	(79.11)	(79.48)	(29.19)	(285)

^*^Statistical significance was compared between OT-infected and TNF-treated HMECs.

P value < 0.05 by Welch’s t-test.

The results of IL-6, CCL2 and CXCL10 secretion from OT-infected HMECs at 8, 16, 24, and 48 hours post-infection were shown in (**Figure 1C**). The IL-6 level slightly increased from 8 h to 48 h post-infection. The CCL2 level moderately increased at 8 h, reached a peak at 16 h and was persistent throughout 48 h post-infection. The CXCL10 level constantly increased from 8 to 24 h post-infection and was persistent at the late stage of infection.

### HMECs Express Pro-Caspase-1 but Lack NLRP3, Pro-Caspase-4, Pro-IL-1β and Caspase-1 Cleavage

**Figure 2:**
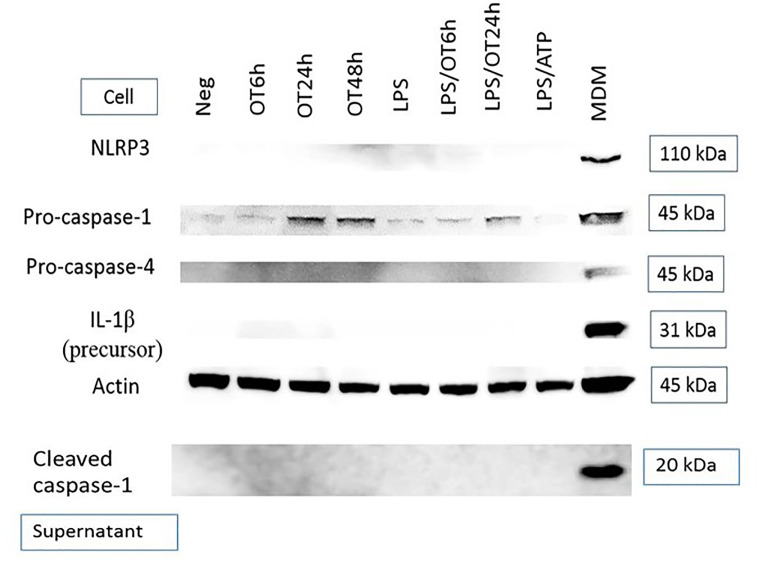
Immunoblot of components associated with inflammasome activation. HMECs were primed or unprimed with LPS (1 µg/ml) for 6 hours before OT infection. Lysates and supernatants were collected at indicated time. LPS-primed and ATP-stimulated HMECs were included as a control. The positive controls were LPS-primed and OT-stimulated monocyte-derived macrophages (MDM, cell lysates) and *Salmonella*-infected MDM (supernatants). Data are representative of three independent experiments.

Inflammasome activation is another hallmark of response to a cytosolic pathogen that is usually described in mononuclear phagocytes including monocytes, macrophages and dendritic cells (Miao et al., 2011; He et al., 2016; Malik and Kanneganti, 2017). Previous studies have demonstrated inflammasome activation and IL-1β secretion from OT-infected monocytes and macrophages (Tantibhedhyangkul et al., 2011; Koo et al., 2012; Tantibhedhyangkul et al., 2013). However, the data of inflammasome activation in endothelial cells during an infection are still limited. We analyzed protein expression of components associated with the inflammasome complex and IL-1β release, including NLRP3, pro-caspase-1, pro-caspase-4 [associated with non-canonical inflammasome activation (Yang et al., 2015)], pro-IL-1β and cleaved caspase-1 (p20). We demonstrated that HMECs expressed only pro-caspase-1 and its expression seemed to be increased by OT infection. In contrast, the expression NLRP3, pro-IL-1β, pro-caspase-4, and cleaved caspase-1 was undetectable (**Figure 2**). The absence of these components correlated with the inability of HMECs to secrete IL-1β. Like LPS-primed HMECs, priming of HMECs with TNF or cytokines from infected monocytes failed to induce these protein components except pro-caspase-1 (Data not shown).

### Upregulation of ICAM-1, E-Selectin and Tissue Factor on HMECs Is Mediated by TNF and Cytokine(s) From Monocytes, Not by OT Infection Per Se

**Figure 3:**
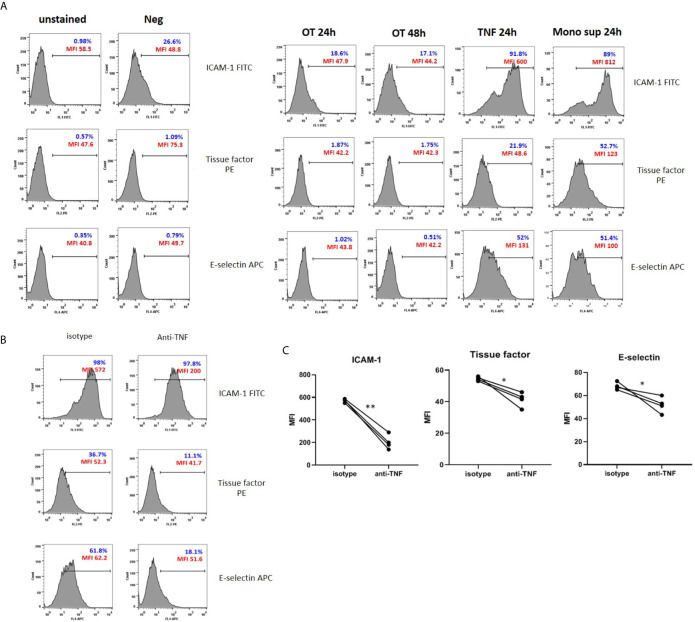
Surface expression of adhesion molecules and tissue factor on HMECs. **(A)** Flow cytometry results of endothelial cell activation shown in histograms. HMECs were infected with OT for 24 and 48 h, stimulated with TNF or monocytes supernatants for 24 h. ICAM-1, tissue factor, E-selectin positive cells were gated from an unstained control. Percentage of positive cells (blue) and mean fluorescent intensity (MFI, red) were shown. Data are representative of three independent experiments. **(B, C)** Effect of TNF secreted from monocytes on EC activation. HMECs were pretreated with either isotype control or anti-TNF antibodies before stimulation with monocyte supernatants. Histograms of surface molecule expression were shown in Figure 3B. Mean fluorescent intensity (MFI) values of four independent experiments were shown in **(C)**. *P value < 0.05; **P value < 0.001.

Since endothelial cell activation is usually induced by inflammatory cytokines TNF and IL-1 (Kilgore et al., 1995; Makó et al., 2010), the absence of cytokine secretion may not induce HMEC activation. We analyzed the surface expression of tissue factor and adhesion molecules, including ICAM-1 and E-selectin on HMECs after OT infection, TNF, or monocyte supernatant stimulation. Low level of ICAM-1 was expressed on unstimulated HMECs. After stimulation, these three markers were upregulated by TNF and monocyte supernatant, but not by OT infection in HMECs (**Figure 3A**). Results of antibody staining on adherent HMECs showed that ICAM-1 and E-selectin were slightly upregulated on HMECs after OT infection. Cell stimulation with either TNF or monocyte supernatant markedly increased all these three markers (**Supplementary Figure 4**).

Since the monocyte TNF level was much higher than IL-1β, we questioned whether TNF is the main cytokine from monocytes that promotes endothelial cell activation. Therefore, we pretreated HMECs with anti-TNF or isotype control before stimulation with monocyte supernatants and analyzed adhesion molecules and tissue factor expression. The expression of ICAM-1, E-selectin, and tissue factor was significantly decreased in condition with anti-TNF, compared to isotype control (**Figures 3B, C** **)**. Thus, TNF is the main cytokine from infected monocytes that induces endothelial cell activation.

## Discussion

Endothelial cells are one of the main targets of OT infection. The complications in rickettsial infections result from vasculitis and vascular injury induced by bacterial invasion into ECs and inflammatory response. However, it is unclear whether the inflammatory response is mediated by ECs or neighboring immune cells such as mononuclear phagocytes. This study showed that ECs produce moderate levels of chemokines and low levels of cytokines (IL-6 and IFN-β) but undetectable levels of proinflammatory cytokines (TNF, IL-1β). Chemokine secretion by ECs was enhanced by TNF and cytokine(s) from infected monocytes. We also showed that upregulation of ICAM-1, E-selectin, and tissue factor in ECs was mediated by cytokine(s), especially TNF, from monocytes, but not by direct invasion of OT into ECs.

Previous studies have shown the chemokine mRNA upregulation in OT-infected HMECs (Cho et al., 2001; Ge et al., 2019; Mika-Gospodorz et al., 2020), but chemokine secretion has not been studied. The effects of cytokines from immune cells on HMECs in OT infection have never been investigated. Evidence suggests that monocytes are the primary target cell, particularly at the early stage of infection (Paris et al., 2012b). Previous studies have shown that monocytes and macrophages produce high levels of inflammatory cytokines TNF and IL-1 (Tantibhedhyangkul et al., 2011; Tantibhedhyangkul et al., 2013). There may be some differences between monocytes and ECs. In this study, we showed that OT mainly induced chemokine secretion without TNF and IL-1β. The low cytokine secretion from ECs may be an immune evasion strategy that prevents early endothelial cell activation and inflammatory response. On the other hand, chemokines (CCL2, CCL5, CXCL10) recruit monocytes and lymphocytes at the infected sites (Viola et al., 2008). These chemokines may be partly beneficial to host defense, but they may attract monocytes from blood to tissues. Consequently, due to the OT tropism for monocytes/macrophages, the accumulation of these target cells may promote bacterial replication and is thus detrimental to the host.

Lack of TNF and IL-1β secretion may be an intrinsic property of HMECs. Our previous study also demonstrated the lack of TLR2 and TLR4 ligand in OT organisms (Tantibhedhyangkul et al., 2017a), which may explain the inadequate cytokine secretion in OT-infected HMECs. Moreover, the lack of NLRP3 expression, which is the crucial receptor for inflammasome activation (Swanson et al., 2019), may explain the inability of HMECs to cleave caspase-1 and secrete IL-1β. IFN-β together with IL-6 is the signature response to cytosolic DNA (Shirota et al., 2006), the putative ligand of cytosolic pathogens (Stetson and Medzhitov, 2006). Our results showed that HMECs secrete IL-6 and IFN-β, but the levels are much lower than monocytes. IL-6 is implicated in the pathogenesis of some infectious diseases such as COVID-19 (McGonagle et al., 2020). Thus, a study of IL-6 in scrub typhus is worth pursuing. Similar to previous studies, we observed that the secretion of IFN-β is persistent at 24 and 48 h post-infection. The positive feedback mechanism of type I IFNs can explain this persistence (Decker et al., 2005). IFN-β subsequently upregulates interferon-stimulated genes, including chemokines such as CCL5 and CXCL10 (Van Boxel-Dezaire et al., 2006), which are upregulated in our study. Since these chemokines attract mononuclear cells, we hypothesize that IFN-β is partly involved in perivascular mononuclear cell infiltration and vasculitis in scrub typhus.

Our focus here is on CCL2 (chemokine for monocytes) and CXCL10 (chemokines for lymphocytes during type 1 immune response). Several cytokines have been shown to upregulate these chemokines. CCL2 (MCP-1) is a key cytokine for monocyte migration, can be upregulated by proinflammatory cytokines (e.g. TNF, IL-1 and IL-6) (Deshmane et al., 2009; Bianconi et al., 2018) and plays an important role in host defense against several intracellular pathogens (Serbina et al., 2008). CXCL10 (IP-10) is upregulated by TNF and interferons (Lee et al., 2013), interacts with CXCR3 expressed on Th1, CD8+ T cells and NK cells to attract these lymphocytes to infected sites (Viola et al., 2008; Metzemaekers et al., 2017). Apart from CXCL10 and CCL5 in this study, other CXCR3 ligands (CXCL9 and CXCL11) and CCL5 ligands (CCL3 and CCL4) were reportedly upregulated in OT-infected cells or in sera of infected animals or humans (Cho et al., 2000; Koh et al., 2004; De Fost et al., 2005; Yun et al., 2005; Tantibhedhyangkul et al., 2011). We hypothesize that proinflammatory cytokines (TNF, IL-1, IL-6) play an essential role in promoting chemokine production during early infection because these inflammatory cytokines are usually decreased at the late phase (De Fost et al., 2005; Tantibhedhyangkul et al., 2013). Then, IFN-β is more critical during late infection because of the persistent kinetics of type I IFN production.

Similar to our study, previous studies have shown that *Rickettsia*-infected human umbilical vein endothelial cell (HUVECs) secrete IL-6, but not TNF or IL-1β. In addition, cell-associated IL-1α is detectable in cell lysates, but not supernatants of rickettsia-infected cells (Kaplanski et al., 1995; Sporn and Marder, 1996). Our study focuses on IL-1β because this cytokine is secreted upon inflammasome activation, contributes to systemic inflammation, and is more widely studied (Dinarello, 2011). On the other hand, IL-1α usually exists as a membrane-bound protein and can be released upon cell death (Malik and Kanneganti, 2018). A previous study has demonstrated that OT-infected ECV304 secrete several cytokines and chemokines including TNF, IL-1β, and IL-6 (Cho et al., 2010). Since ECV304 is known to cross-contaminate with a urinary bladder carcinoma cell line (Dirks et al., 1999; Brown et al., 2000), the response of this cell line may not truly represent that of endothelial cells. Although studies using different endothelial cell origins such as transformed immortalized HMECs and mouse microvascular endothelial cells have demonstrated NLRP3 inflammasome activation and IL-1β release following different stimuli (Xia et al., 2014; Wang et al., 2016; Shrivastava et al., 2020), our results failed to discover these findings. This discrepancy may be due to the differences in endothelial cell origins, culture conditions and the stimuli. In addition, the expression levels of NLRP3 and cleaved caspase-1 in our primary HMECs may be lower than the detection limit of Western blotting in our studies.

**Figure 4:**
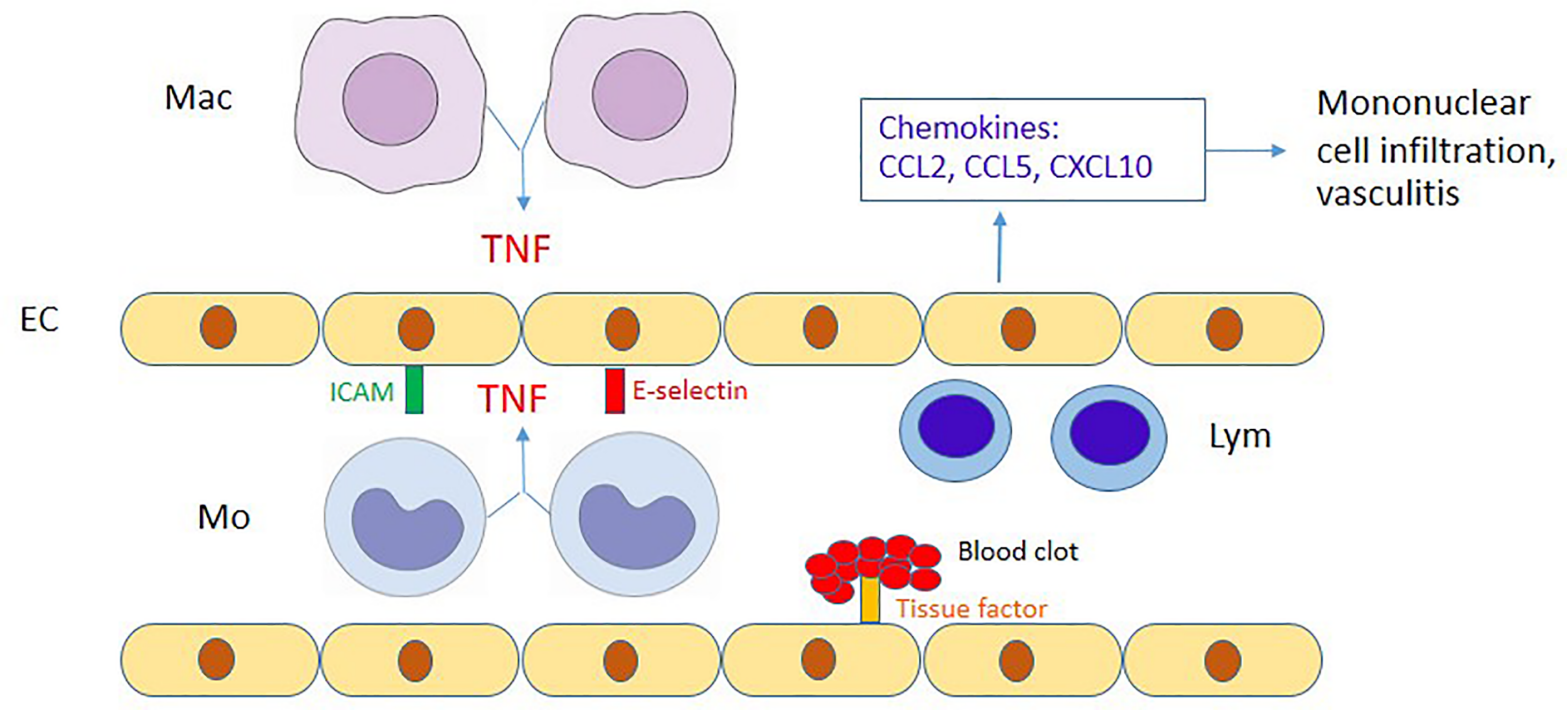
Proposed diagram of the interaction between endothelial cells and monocytes/macrophages. During OT infection, endothelial cells (EC) are activated by TNF from OT-infected monocytes (Mo)/macrophages (Mac), upregulate adhesion molecules (ICAM-1 and E-selectin) and produce chemokines to attract mononuclear cells. CCL2/CCL5 and CCL5/CXCL10 attract monocytes and lymphocytes (Lym), respectively. The immune-mediated mechanisms may contribute to perivascular mononuclear cell infiltration and vasculitis in scrub typhus patients. Moreover, tissue factor upregulated by TNF may contribute to disseminated intravascular coagulation during scrub typhus.

Previous studies have demonstrated that soluble EC activation markers are detectable in scrub typhus patients (Paris et al., 2012a). In addition, these endothelial adhesion molecules were reportedly upregulated in mouse models of scrub typhus (Soong et al., 2017; Trent et al., 2020). However, EC activation mechanisms in scrub typhus are still unclear. We showed that OT infection barely induced upregulation of adhesion molecules (ICAM-1 and E-selectin), but cytokines from monocytes are the primary inducer of EC activation. Among cytokines from monocytes, TNF is likely to play a significant role in EC activation. Lack of early adhesion molecule expression on OT-infected HMECs *per se* may be an immune evasion strategy of OT organisms to prevent lymphocyte migration and evade early lymphocyte killing of infected cells. However, these adhesion molecules and chemokine expression which are upregulated at the later phase of infection by TNF from infected monocytes may recruit mononuclear leukocytes and amplify more inflammation during OT infection. Tissue factor is well known to be upregulated in cardiovascular disease inflammation (Witkowski et al., 2016), but is not widely studied in infectious diseases. We also showed that ECs upregulated surface tissue factor expression following stimulation with monocyte cytokine(s). In contrast to OT infection in this study, a previous study showed that *Rickettsia rickettsii* infection without exogenous TNF treatment was sufficient to upregulate tissue factor expression on HUVECs (Teysseire et al., 1992). This discrepancy may be due to the differences in organisms and host cells. The upregulation of tissue factor on the EC cell membrane may be involved in blood clotting, coagulopathy and disseminated intravascular coagulation (DIC) in scrub typhus patients (Lee et al., 2017). The proposed interaction between endothelial cells and monocytes/macrophages is summarized in **Figure 4**.

In conclusion, we demonstrated the interaction between monocytes and endothelial cells. Cytokines from infected monocytes are the key factor that induces endothelial cell activation and response characterized by upregulation of ICAM-1, E-selectin and tissue factor as well as chemokines for mononuclear cell infiltration. After OT infection, the inadequate cytokine response of endothelial cells may be a subversion strategy to evade host early inflammation and promote bacterial growth. The upregulation of tissue factor may be one mechanism involved in DIC in severe cases of scrub typhus. Further studies are required to clarify this pathogenesis in human scrub typhus patients.

## Data Availability Statement

The raw data supporting the conclusions of this article will be made available by the authors, without undue reservation.

## Ethics Statement

The studies involving human participants were reviewed and approved by Siriraj Institutional Review Board. Written informed consent for participation was not required for this study in accordance with the national legislation and the institutional requirements.

## Author Contributions 

WT conceived and designed the study. WT, SM, AL, and CB performed the experiments and analyzed the data. WT wrote the first draft of manuscript. All authors contributed to the article and approved the submitted version.

## Funding

This research project was supported by Faculty of Medicine Siriraj Hospital, Mahidol University, Grant Number (IO) R015832034. The funders had no role in the design of the study; in the collection, analyses, or interpretation of data; in the writing of the manuscript, or in the decision to publish the results.

## Conflict of Interest

The authors declare that the research was conducted in the absence of any commercial or financial relationships that could be construed as a potential conflict of interest.
