# Supplementary material for: Endothelial Activation in Orientia tsutsugamushi Infection Is Mediated by Cytokine Secretion From Infected Monocytes
Source: Front Cell Infect Microbiol. 2021 Jul 22;11:683017. doi: 10.3389/fcimb.2021.683017 (PMC8340038; doi:10.3389/fcimb.2021.683017)
Supplement: Supplementary file 1 [file DataSheet_1.pdf]

Methods and Results

Supplementary Fig. 1 Intracellular localization of OT in monocytes (A, B) and HMECs (C, D) at 1 hour and 18 hours post-infection.

Monocytes and HMECs grown on coverslips were infected with OT at an MOI of 10:1 for 1 hour. After washing, infected cells were fixed with 3% paraformaldehyde for 15 min or maintained in culture medium for 18 hours before fixation. Cells were permeabilized with 0.1% Triton-X for 10 min before immunofluorescence staining using purified IgG from pooled human sera of scrub typhus patients and mouse monoclonal anti-LAMP-1 (lysosome-associated membrane protein, Biolegend). Secondary antibodies were DyLight 550-labeled anti-human IgG (Abcam) and HiLyte Fluor 488-labeled anti-mouse IgG (AnaSpec). Stained cells were analyzed using ZEISS LSM 800 confocal microscope. Images were processed using ZEN lite 3.3 (blue edition) software (Carl Zeiss).

Supp. Fig. 1 A and B showed the representative results from two monocytes (for each time point). By 1 hour after infection, OT organisms were readily internalized by monocytes (Fig. 1A 3D image) and colocalized with LAMP-1 (late phagosome/ lysosome marker). At 18 hours post-infection, most OT organisms were no longer colocalized with LAMP-1 (Fig. 1B), indicating the cytosolic localization.

Similar to monocyte findings, OT were internalized by HMECs within 1 hour after infection and colocalized with LAMP-1 (Fig. 1C). Since the expression of LAMP-1 is more concentrated in perinuclear region, the OT and LAMP-1 colocalization was more obvious in perinuclear area than at the cell periphery. Similar to the findings from a previous study (Kim SW et al., 2001), most OT organisms moved to perinuclear region and no longer colocalized with LAMP-1 (Fig. 1D).
