# Supplementary material for: Endothelial Activation in Orientia tsutsugamushi Infection Is Mediated by Cytokine Secretion From Infected Monocytes
Source: Front Cell Infect Microbiol. 2021 Jul 22;11:683017. doi: 10.3389/fcimb.2021.683017 (PMC8340038; doi:10.3389/fcimb.2021.683017)
Supplement: Supplementary file 2 [file DataSheet_2.pdf]

A

IL-1b (pg/ml)	OT	LPS/OT
6 h	ND	138 (23)
24 h	4 (0.5)	125 (30)

LPS/ATP (1 h)
610 (80)

B

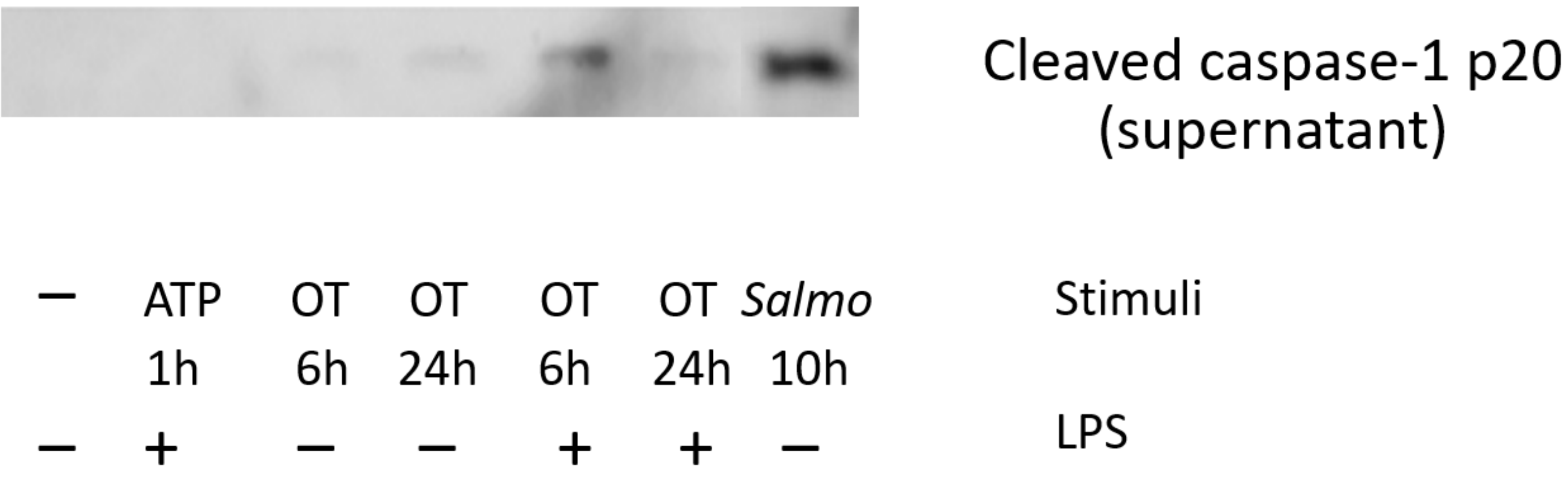

Suppl. Fig. 2 Inflammasome activation determined by IL-1β secretion and cleaved caspase-1 release from macrophages.

A. Monocyte-derived macrophages were primed or unprimed with LPS (100 ng/ml) for 5 h, stimulated with OT for 6 h and 24 h or ATP for 1 h. IL-1β secretion was analyzed by ELISA. Data are expressed as mean ± SEM of three experiments.

B. Cleaved caspase-1 in supernatants were analyzed by Western blot.

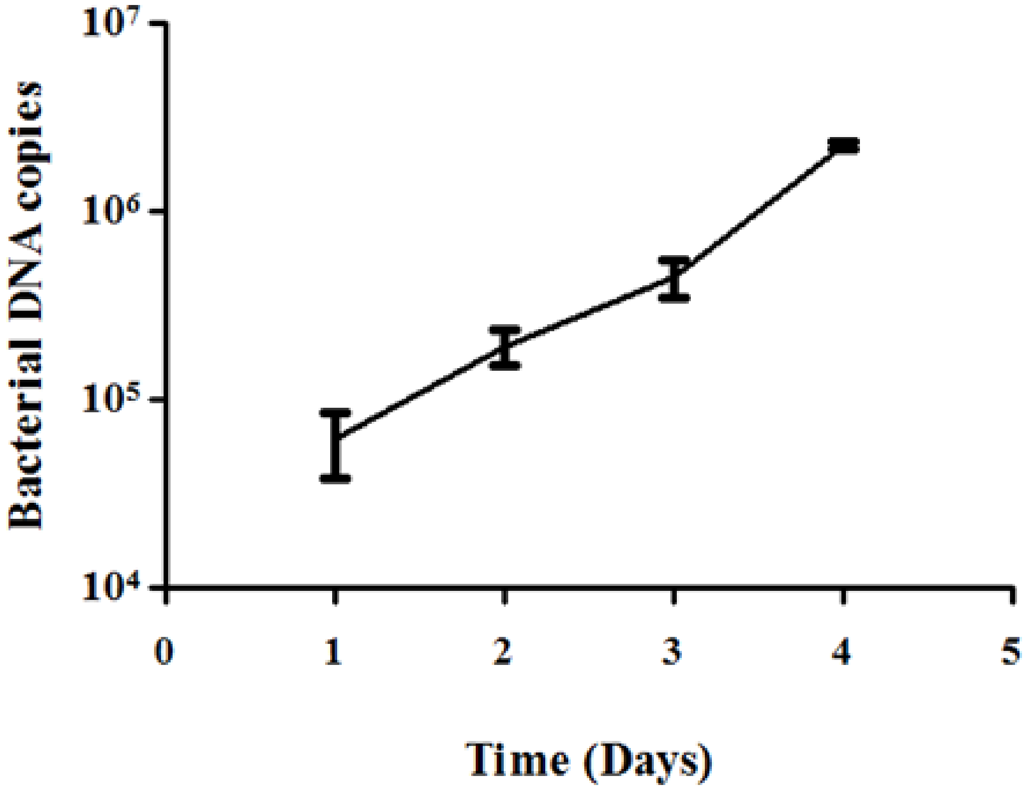

Suppl Fig. 3 Growth of OT in HMECs. HMECs were infected with OT an MOI of 5 and maintained for 4 days. Bacterial DNA copies were determined by qPCR targeting gene encoding 47 kDa membrane protein (Tantibhedhyangkul et al., 2017b)

Doubling time is about 14 hours.

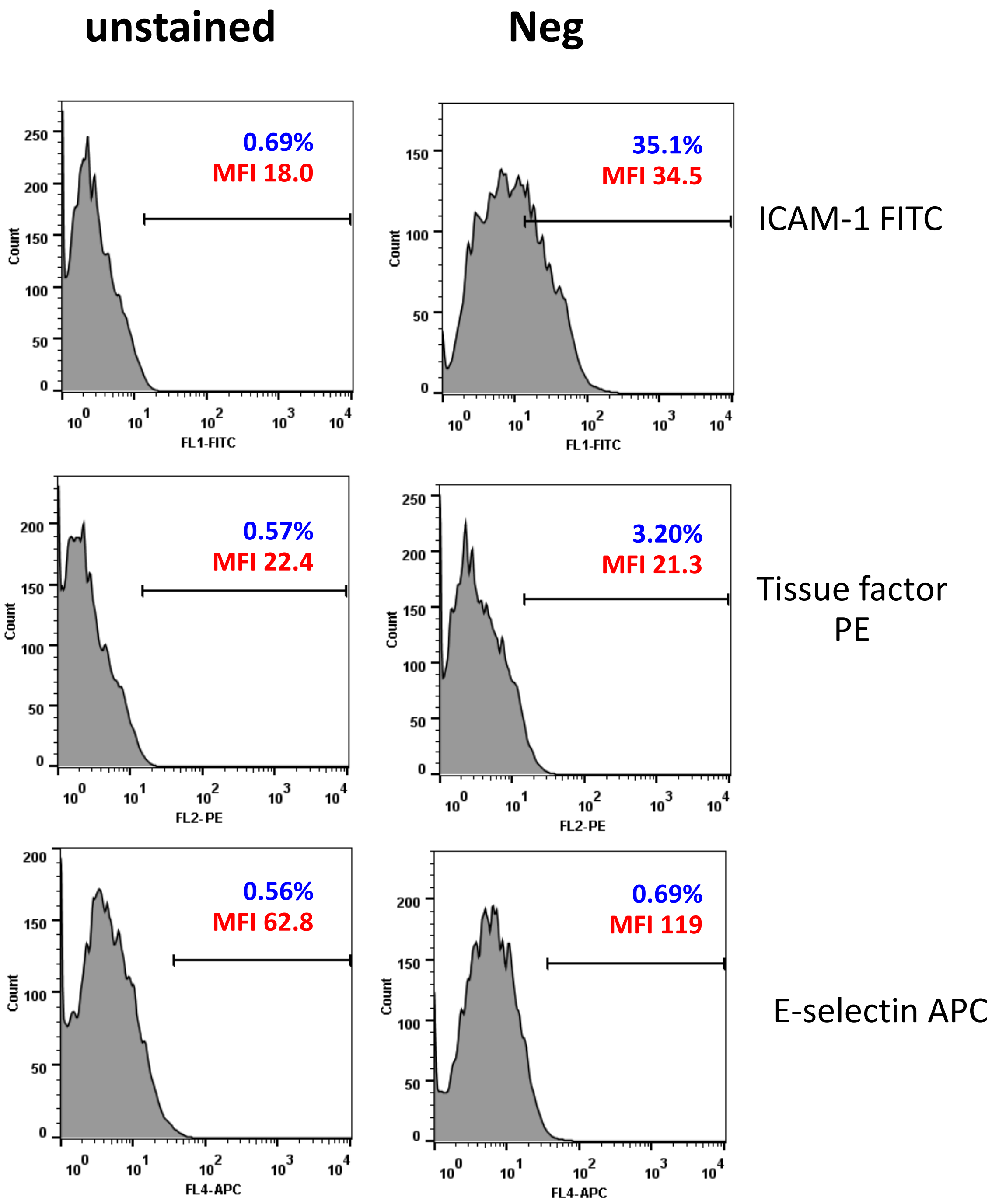

Suppl. Fig. 4

Adherent HMECs were stained with FITC anti-ICAM-1, PE-anti-tissue factor and APC anti-E-selectin. After antibody staining, cells were washed, detached with 0.5 mM EDTA in PBS with 0.5% human serum albumin. Cells were pelleted, washed, fixed in 2% paraformaldehyde and analyzed by flow cytometry.

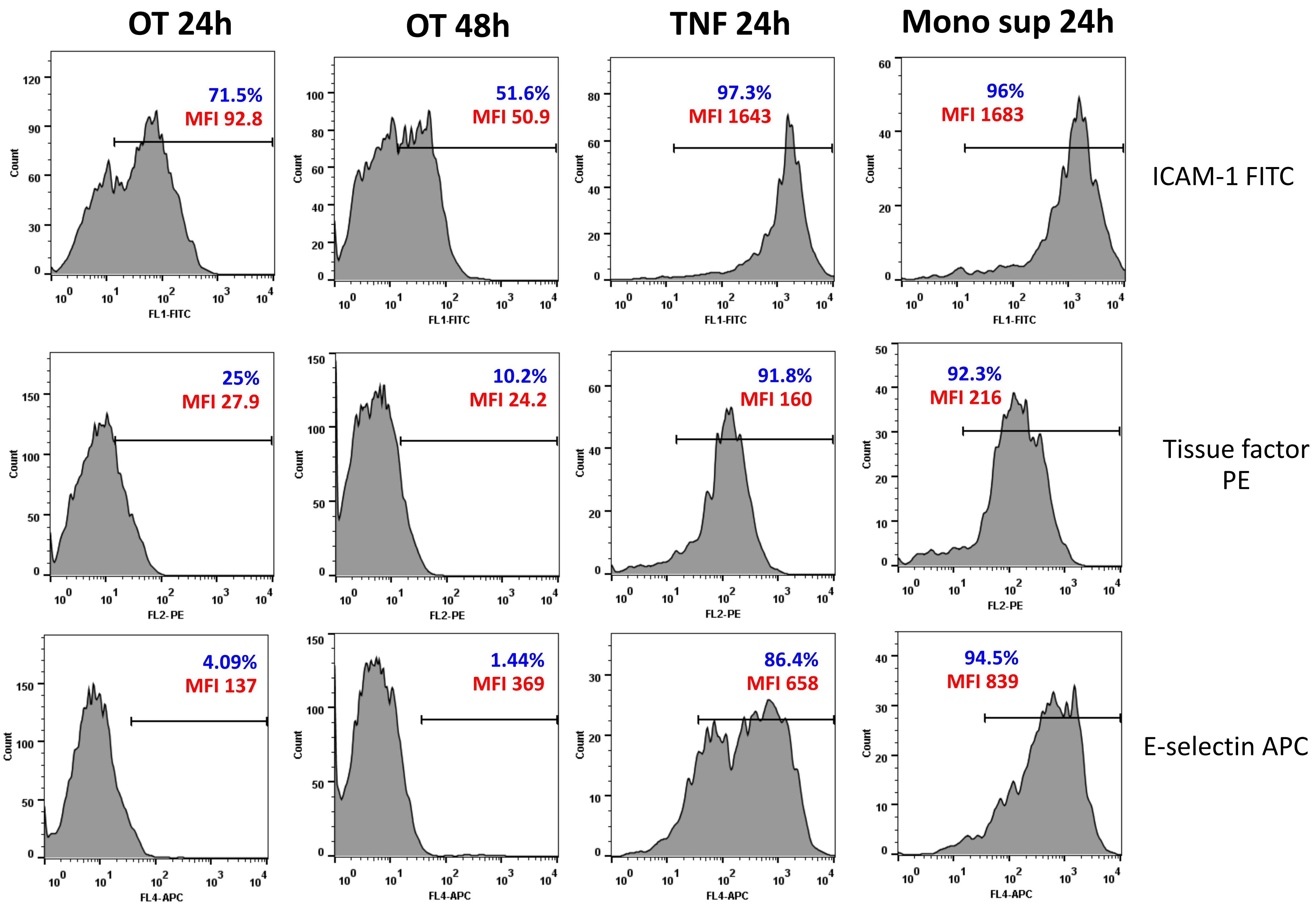

OT 24h
OT 48h
TNF 24h
Mono sup 24h
71.5%
MFI 92.8
51.6%
MFI 50.9
97.3%
MFI 1643
96%
MFI 1683
ICAM-1 FITC
25%
MFI 27.9
10.2%
MFI 24.2
91.8%
MFI 160
92.3%
MFI 216
Tissue factor PE
4.09%
MFI 137
1.44%
MFI 369
86.4%
MFI 658
94.5%
MFI 839
E-selectin APC
Count
FL1-FITC
FL2-PE
FL4-APC
