# Supplementary figures and images for: Endothelial Activation in Orientia tsutsugamushi Infection Is Mediated by Cytokine Secretion From Infected Monocytes
Source: Front Cell Infect Microbiol. 2021 Jul 22;11:683017. doi: 10.3389/fcimb.2021.683017 (PMC8340038; doi:10.3389/fcimb.2021.683017)

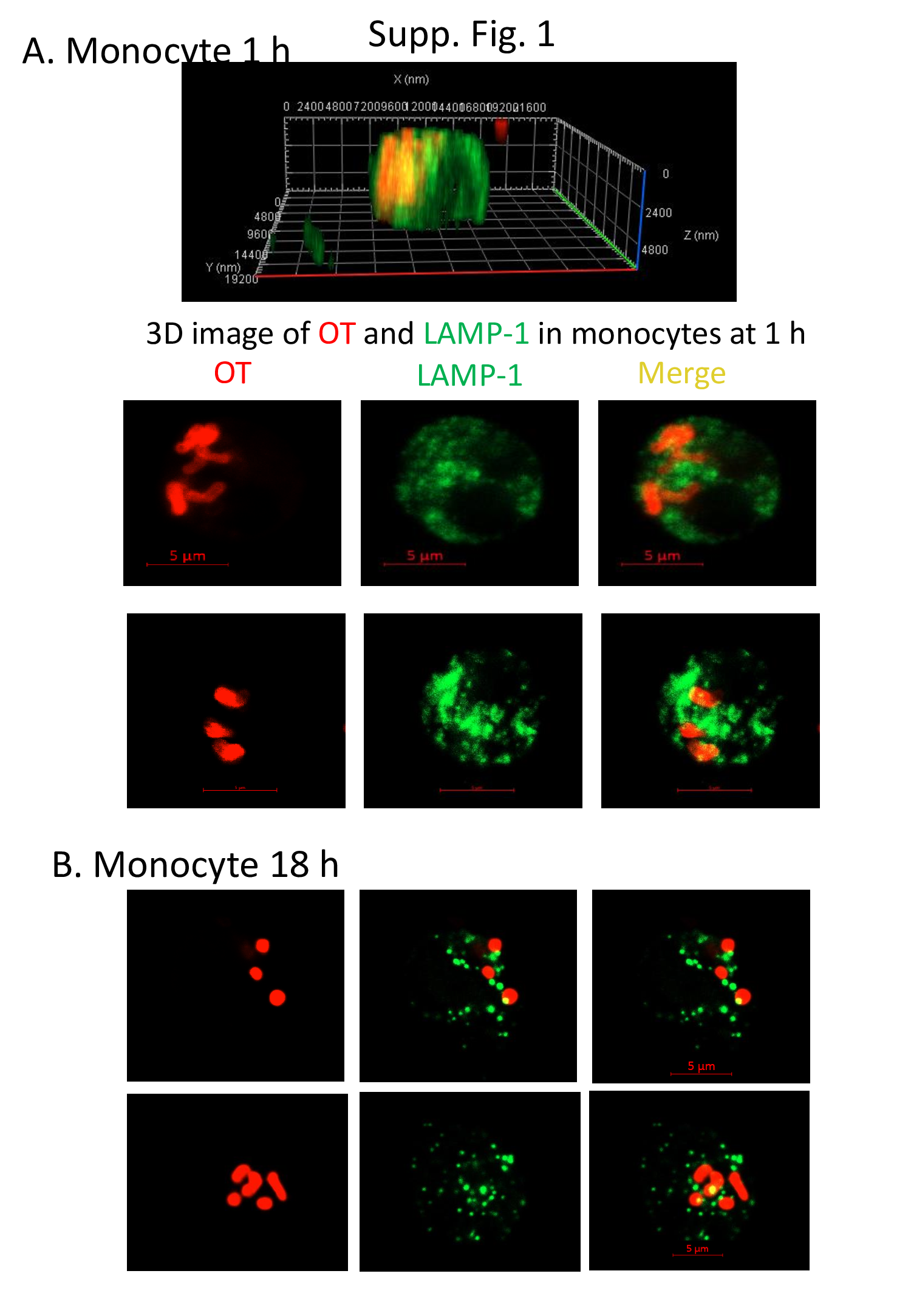

Supplement: Supplementary file 3 [file Image_1.tiff]

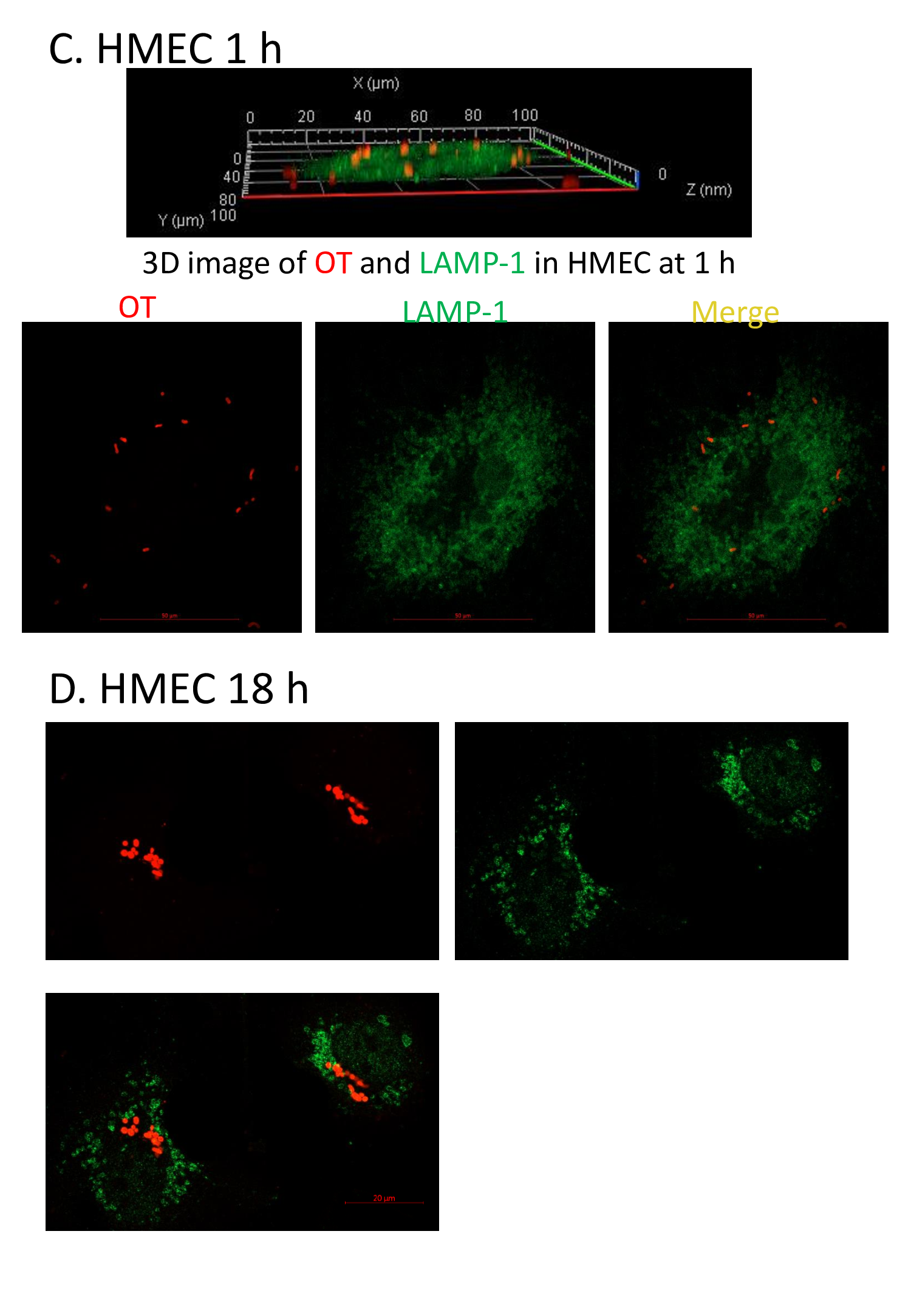

Supplement: Supplementary file 4 [file Image_2.tiff]
